# LONG-TERM ASSOCIATION OF DIABETES CONTROL WITH SELF-REPORTED VISION AND HEARING

**DOI:** 10.1093/geroni/igae098.0387

**Published:** 2024-12-31

**Authors:** Mark Espeland, Michael Walkup, Lynne Wagenknecht, Kathleen Hayden, Charles Semelka, Rena Wing, Tara Beckner, Denise Houston

**Affiliations:** Wake Forest University School of Medicine, Winston-Salem, North Carolina, United States; Wake Forest University School of Medicine, Winston-Salem, North Carolina, United States; Wake Forest University School of Medicine, Winston-Salem, North Carolina, United States; Wake Forest University School of Medicine, Winston-Salem, North Carolina, United States; Wake Forest University School of Medicine, Winston-Salem, North Carolina, United States; Miriam Hospital, Providence, Rhode Island, United States; Wake Forest University School of Medicine, Winston-Salem, North Carolina, United States; Wake Forest University School of Medicine, Winston Salem, North Carolina, United States

## Abstract

Older adults with type 2 diabetes are at increased risk for vision and hearing loss. We examined whether better control of glycosylated hemoglobin (HbA1c), blood pressure, and weight was associated with better self-reported vision and hearing acuity among older adults with type 2 diabetes and overweight or obesity. The Look AHEAD Aging study administered a standardized questionnaire to N=1,008 individuals, mean (SD) age 77.8 (5.7) years, who had been enrolled in the Look AHEAD trial of intensive lifestyle intervention (ILI). We examined how mean levels of HbA1c, systolic blood pressure (SBP), and body mass index (BMI) over the prior 10 years were associated with markers of vision and hearing acuity, controlling for age, gender, and diabetes duration. Hearing and vision deficits were assessed by self-report of fair, poor, or worse abilities. Multisensory deficits were defined as reported deficits for both vision and hearing. Relatively better 10-year control of HbA1c was associated with less prevalent vision deficits (OR=0.72 per IQR lower HbA1c, p=0.002) and with less prevalent multisensory deficits (OR=0.65 per IQR lower HbA1c, p=0.002). Better 10-year SBP control was also associated with less prevalent vision deficits (OR=0.72 per IQR lower SBP, p=0.005). Prior random assignment to ILI and 10-year BMI levels were not associated with self-reported vision. HbA1c, SBP, BMI, and intervention assignment were not significantly associated with hearing acuity. Long-term control of HbA1c and SBP are important to maintaining vision in older individuals with type 2 diabetes. HbA1c control may also reduce the prevalence of multisensory deficits.

